# Novel Insight Into the Relationship of Vitamin D Hydroxylase and Vitamin D With Obesity in Patients With Type 2 Diabetes Mellitus

**DOI:** 10.7759/cureus.49950

**Published:** 2023-12-05

**Authors:** Maha M Bakhuraysah, Amal F Gharib, Asmaa F Hassan, Ghadi K Al Harthi, Raghad F Al Thobaiti, Maha M Al Adwani, Ahmed D Alharbi, Abdullah S Alzahrani, Khwaimsah M Alsubei, Rana F Al-Asiri

**Affiliations:** 1 Clinical Laboratory Sciences, College of Applied Medical Sciences, Taif University, Taif, SAU; 2 Physiology, College of Medicine, Taif University, Taif, SAU; 3 Laboratory, King Faisal Medical Complex, Taif, SAU; 4 Research Center, King Faisal Medical Complex, Taif, SAU

**Keywords:** vitamin d hydroxylase, lipid profile, obesity, diabetes mellitus, vitamin d

## Abstract

Background: In patients with type 2 diabetes mellitus (T2DM) and obesity, there is often a vitamin D deficiency, which is crucial for many physiological processes. The enzyme 25-D hydroxylase activates vitamin D, and its status has been linked to glucose and lipid metabolism in these patients. This study investigates the correlation between the levels of 25-D hydroxylase and 25-hydroxyvitamin D, and their impact on glucose and lipid metabolism in Saudi patients with T2DM and obesity.

Methods: This study involved 150 Saudi participants (ages 35-70) of both genders from King Faisal Medical Complex in Taif. The participants were divided into control, type 2 diabetic, and obese diabetic patient groups, with 50 subjects in each group. Serum levels of 25(OH) vitamin D and 25-D hydroxylase were measured using ELISA. In contrast, an automatic analyzer for chemistry tests was used to measure fasting blood glucose (FBG), aspartate transaminase (AST), alanine transaminase (ALT), and lipid profile levels. Hemoglobin A1c (HbA1c)% was analyzed using the automatic glycosylated hemoglobin analyzer. In addition, the body mass index (BMI) value was calculated using the equation (BMI = weight in kilograms/height in meters squared).

Results: In obese with T2DM and T2DM patient groups, there were significant increases (p < 0.0001) in BMI, FBG, HbA1c%, total cholesterol (TC), triglycerides (TG), low-density lipoprotein cholesterol (LDL-C), AST, and ALT levels and significant decreases (p < 0.0001) in 25(OH) vitamin D, 25-D hydroxylase, and high-density lipoprotein cholesterol (HDL-C) levels compared to the control group. There were significant positive correlations between vitamin D with 25-D hydroxylase and HDL-C and negative correlations with HbA1c%, FBG, TC, TG, LDL-C, ALT, AST, and BMI in the studied patient groups.

Conclusions: This study shows that lower levels of the enzyme 25-D hydroxylase are linked to reduced vitamin D levels in people with T2DM and obese diabetic patients. Additionally, there was a notable correlation between vitamin D levels and BMI, lipid profile, FBG, HbA1c%, AST, and ALT levels.

## Introduction

Type 2 diabetes mellitus (T2DM) is a chronic metabolic disorder caused by the pancreas' failure to secrete sufficient insulin or the body's inability to properly use the insulin it produces [1]. Approximately seven million people in Saudi Arabia have diabetes, and more than three million have pre-diabetes, making it the country with the second-highest rate of the disease in the Middle East and the seventh-highest worldwide. Its prevalence in Saudi Arabia has been estimated at approximately 32.8%. Furthermore, the predicted prevalence is 40.37% in 2025 and 45.36% in 2030 [2]. T2DM risk is raised with family history, age, obesity, and lack of physical activity. Women with prior gestational diabetes mellitus (GDM) and individuals with impaired glucose tolerance (IGT) or impaired fasting glucose (IFG) are also at risk of developing T2DM [3]. Furthermore, T2DM patients are more exposed to different complications, including macrovascular diseases (heart attacks, coronary artery disease, strokes, hypertension, and hyperlipidemia), microvascular diseases (retinopathy, neuropathy, and nephropathy), and cancers [4]. Recently, vitamin D deficiency has sparked widespread interest as a risk factor for developing T2DM [5]. According to epidemiological studies, vitamin D deficiency affects up to one billion people worldwide [6]. A significant 25-D hydroxylase enzyme (CYP2R1) has recently been identified as contributing to hereditary types of vitamin D deficiency [7]. The Saudi population has a high prevalence of low vitamin D levels, which is thought to be caused by excessively high cutoff values for serum 25-hydroxyvitamin D [25(OH) D]. Other than nutrition and sunlight, various things affect vitamin D levels. Saudis had a higher prevalence of hypovitaminosis D than non-Saudis who lived in the same areas and likely consumed a similar diet, and this may be because of genetic factors [8].

Vitamin D is essential for the health of bones and muscles and for the immune system to operate normally. Consequently, vitamin D helps boost defenses against illnesses, including cancer and autoimmune immunological disorders [9]. In addition, vitamin D has been shown to impact insulin function by either enhancing insulin responsiveness for glucose transport by stimulating the expression of insulin receptors or indirectly regulating extracellular calcium levels, which are essential for insulin-mediated processes in insulin-responsive tissues such as skeletal muscles and adipose tissues [10]. There is substantial evidence supporting a link between vitamin D and insulin secretion, which is attributed to the presence of vitamin D receptors (VDR) in pancreatic β cells and vitamin D binding proteins (DBP) in pancreatic tissue. The CYP2R1 gene is associated with 25(OH)D serum levels, encoding the hepatic 25-D hydroxylase that converts parent calciferol into 25(OH)D. Obese individuals have lower circulating concentrations of 25(OH)D than their race, socioeconomic status, and age-matched peers, indicating that vitamin D homeostasis is altered [10]. Aside from the difference in baseline circulating 25(OH)D concentrations, obese subjects have lower increases in serum 25(OH)D levels after being supplemented with vitamin D2 (ergocalciferol) or vitamin D3 (cholecalciferol). Although the cause of this difference is unknown, one possible explanation is that excess adipose tissue sequesters vitamin D, reducing the availability of the substrate for 25-D hydroxylation in the liver. Another mechanism for decreased 25(OH)D production could be decreased expression of CYP2R1 because animal studies indicated that the expression of many hepatic P450 enzymes (i.e., CYPs) is altered in the context of obesity [11]. These changes in CYP expression reflect a chronic inflammatory process induced by obesity that involves both innate and acquired immunity and is associated with significant increases in circulating cytokines. Recent genome-wide association studies (GWASs) have identified the CYP2R1 gene locus as a significant determinant of serum 25(OH)D levels, raising the possibility that CYP2R1 enzyme activity variations may contribute to inter-individual variation in vitamin D homeostasis. Studies have reported that obesity due to a high-fat diet leads to decreased hepatic CYP2R1 mRNA abundance and decreased 25(OH)D conversion [11,12]. Therefore, this study aimed to investigate the relationship between 25-D hydroxylase and 25(OH)D status with glucose and lipid metabolism in obese type 2 diabetic Saudi patients.

## Materials and methods

The current research is a case-control study conducted from August 2022 to June 2023 in the Department of Laboratory Sciences, College of Applied Medical Sciences at Taif University. This study includes 150 Saudi adults of both genders, aged between 35-70 years, attending the Diabetic Clinic at King Faisal Medical Complex, Taif City, enlisted for the study. They were divided into three groups: the control group, T2DM patients, and obese diabetic patients. Participants were assigned to their groups based on the following inclusion and exclusion criteria. Inclusion Criteria: The control group comprised healthy adults with no clinical or laboratory evidence of diabetes mellitus, obesity, or any other diseases. They were matched in age and gender with the patient groups. Diabetic patients included adults with T2DM, based on the American Diabetes Association's (2014) diagnostic criteria. Obese patients included individuals with a BMI of 30 kg/m^2 or higher. Exclusion Criteria: Patients with type 1 diabetes, metabolic syndrome, malabsorptive states, or gestational diabetes were excluded. Individuals with renal diseases, hepatic diseases, chronic illnesses, malignancy, or any blood diseases were also excluded. Patients who had received vitamin D or calcium supplements for less than three months and non-Saudi patients were not included in the study.

Study experiments and methods

Peripheral fasting blood samples of all participants, both patients and control subjects, were collected in plain tubes, and serum was then separated. These serum samples were then stored at -20°C until analyzed. The serum lipid profile, comprising total cholesterol (TC), triglycerides (TG), low-density lipoprotein cholesterol (LDL-C), and high-density lipoprotein cholesterol (HDL-C), along with fasting blood glucose and liver enzymes (aspartate transaminase [AST] and alanine transaminase [ALT]), were measured using the Cobas c 501 automatic analyzer for chemistry tests. Serum 25(OH) vitamin D and 25-D hydroxylase levels in all participants were estimated using the "Double Antibody Sandwich" enzyme-linked immunosorbent assay (ELISA) technique (MyBioSource, Cat.No. MBS268910, and Cat.No. MBS167202). The human 25-hydroxy vitamin D (25(OH)D) ELISA kit is designed for the quantitative detection of human serum, cell culture supernatant, or plasma. The process involves using a pre-coated antibody, specifically an anti-Human 25(OH)D monoclonal antibody, and a biotinylated polyclonal detection antibody. Control and patient serum samples, along with the biotinylated antibodies, were added to the ELISA 96-well plate. The wells were then washed with either PBS or TBS. Avidin-peroxidase conjugates were added to the wells, followed by a thorough wash with PBS or TBS, and then a TMB substrate was introduced for color development. The peroxidase activity of TMB resulted in a blue product, which changed to yellow upon adding the stop solution (Color Reagent C). The intensity of the color correlates directly with the concentration of the target analyte in the sample. Absorbances were measured at 450 nm using a microplate reader (Bio-Rad Laboratories, California). The human vitamin D 25-hydroxylase (CYP2R1) ELISA sandwich kit, designed for accurate and quantitative detection of human vitamin D 25-hydroxylase (CYP2R1) in various biological samples, follows a similar ELISA principle. The 96-well assay plate pre-coated with Human CYP2R1 antibodies received control and patient serum samples containing CYP2R1. A biotinylated Human CYP2R1 antibody was added, followed by streptavidin-horse radish peroxidase (HRP), binding to the biotinylated CYP2R1 antibody. After incubation, any unbound Streptavidin-HRP was removed during a washing step. The substrate solution added subsequently led to the development of color proportional to the amount of Human CYP2R1 present in the sample. The reaction was stopped with an acidic stop solution, and the absorbances were again measured at 450 nm using a microplate reader (Bio-Rad Laboratories).

Another part of the blood samples was collected in EDTA-containing tubes for hemoglobin A1c (HbA1c)% estimation using the automated glycosylated hemoglobin analyzer (Bio-Rad). The body mass index (BMI) value was calculated using the formula (BMI = weight in kilograms/height in meters squared). The study was approved by the research ethical committee of the King Faisal Medical Complex in Taif (2023-B-6), and informed consent was obtained from each participant after the study's purpose and procedures were clearly explained.

Data analysis method

Our data for this research were analyzed by using GraphPad Prism version 8. The one-way analysis of variance (ANOVA) was used between different variables, followed by a post hoc test for comparisons between multiple groups. We used Pearson's correlation coefficient to evaluate the relationship between vitamin D and other parameters in all patient groups. P-values were considered statistically significant at <0.05.

## Results

**Figure 1:**
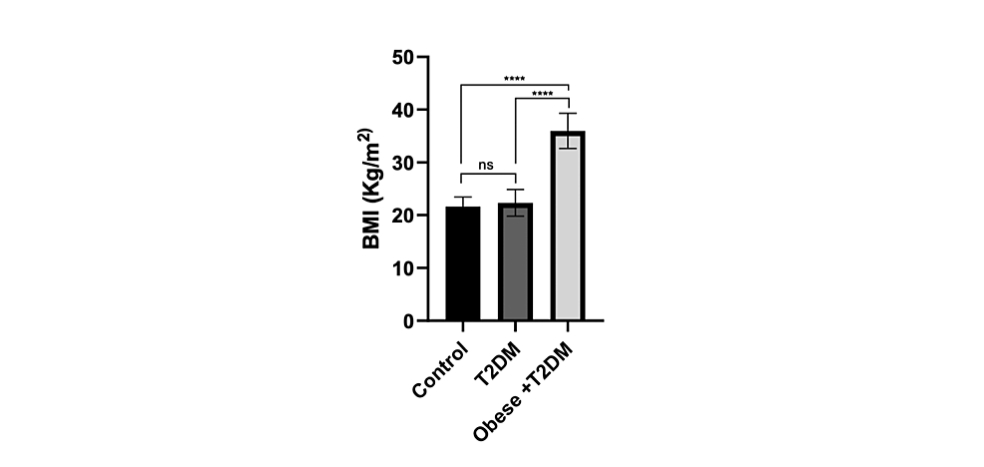
Comparison of BMI in control patient groups. (ns=non-significant, **** p < 0.0001). Data are means ± SD. TD2M: type 2 diabetes mellitus.

Results of this study showed that the mean ± SD of BMI in the T2DM and control groups were non-significantly varied. In the obese + T2DM group, however, it was significantly increased (p < 0.0001) compared to the T2DM and control groups, as shown in Figure 1.

**Figure 2:**
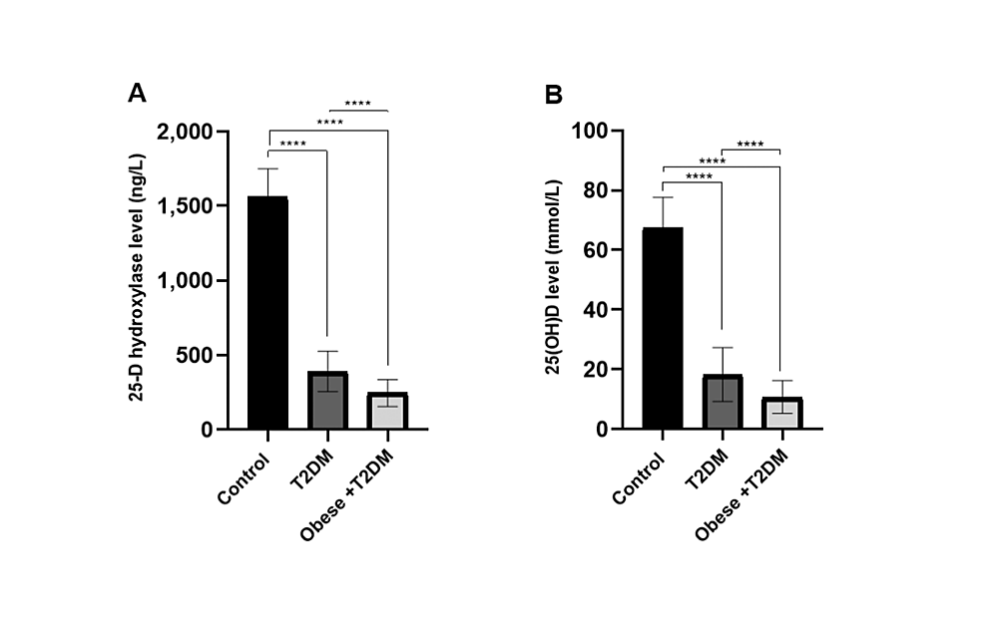
(A) Serum levels of 25-D hydroxylase (ng/L) in control, T2DM, and obese +T2DM groups. (B) 25(OH) vitamin D (mmol/L) and in control and patient groups. (****p < 0.0001) (means ± SD). T2DM: type 2 diabetes mellitus.

Serum levels of 25-D hydroxylase in the T2DM and obese + T2DM groups were significantly decreased (p < 0.0001) compared to the control group. When comparing the T2DM and obese groups, the levels showed a significant decrease (p < 0.0001) in the obese + T2DM group (Figure 2A). Also, serum 25(OH) vitamin D levels in both patient groups were significantly decreased (p < 0.0001) compared to the control group. Among the obesity + T2DM patients' group, the levels were significantly lower (p < 0.0001) compared to the T2DM patients' group (Figure 2B).

**Figure 3:**
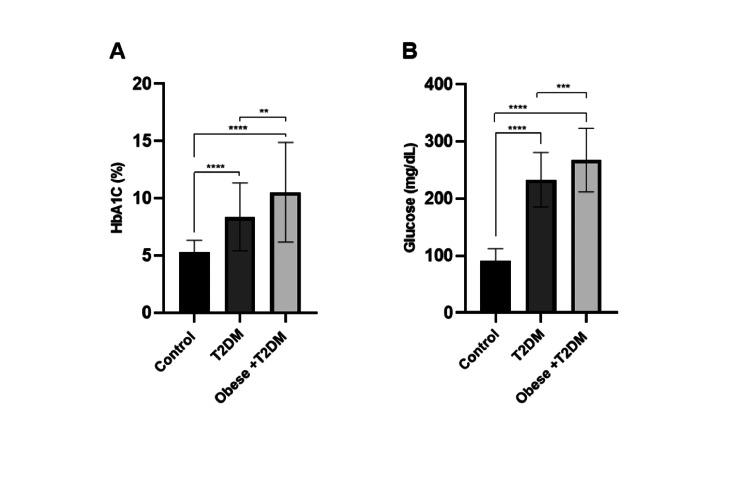
(A) Comparison of HbA1c% in control and patient groups. (B) Comparison of FBG (mg/dL) levels in control and patient groups (**** p < 0.0001, *** p = 0.0005, ** p = 0.002). Data are means ± SD. T2DM: type 2 diabetes mellitus.

Figure 3A-B shows the HbA1c% and FBG levels among the control, T2DM, and obese + T2DM patient groups. The mean ± SD of HbA1c% in both the T2DM and obese + T2DM groups was significantly higher (p < 0.0001) than in the control group. HbA1c% was significantly increased in the obese group (p = 0.002) compared to the T2DM patients' group (Figure 3A). The mean ± SD of FBG levels in the T2DM and obese + T2DM groups was significantly higher (p < 0.0001) compared to the control group. There were significantly increased FBG levels in the obese + T2DM group (p = 0.0005) compared to the T2DM patients' group (Figure 3B).

**Figure 4:**
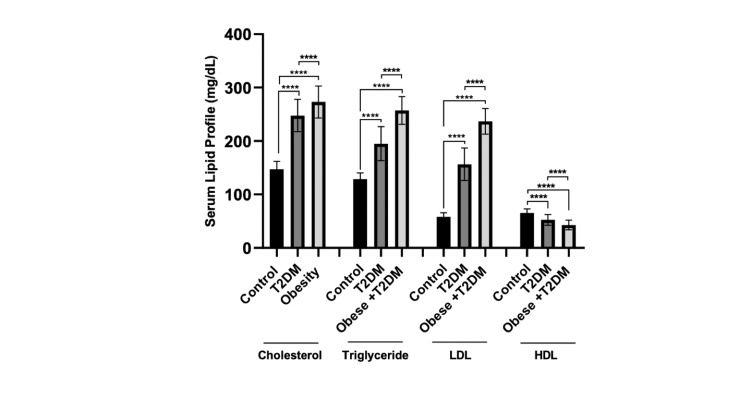
Serum levels (means ± SD) of lipid profile (mg/dL) in control and patient groups (**** p < 0.0001). T2DM: type 2 diabetes mellitus.

Figure 4 shows that the serum levels of TC were significantly higher (p < 0.0001) in both the T2DM and obese + T2DM groups as compared to the control group. Also, serum TG and LDL-C levels were significantly higher (p < 0.0001) in both patients’ groups compared to the control group. The levels of these parameters were significantly higher in the obese + T2DM patients than in the T2DM patients (p < 0.0001). Conversely, serum levels of HDL-C were significantly decreased (p < 0.0001) in the T2DM and obese + T2DM groups compared to the control group. The obese + T2DM group had significantly lower HDL-C levels (p < 0.0001) compared to the T2DM group.

**Figure 5:**
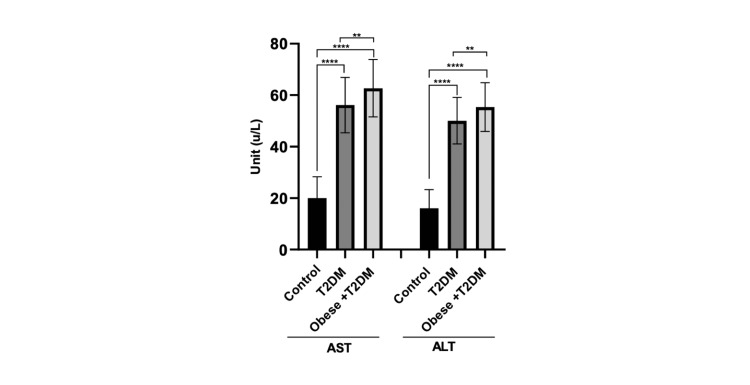
Serum levels (means ± SD) of AST and ALT (u/L) in control and patient groups (**** p < 0.0001, ** p = 0.0056). T2DM: type 2 diabetes mellitus, AST: aspartate transaminase, ALT: alanine transaminase.

Serum levels of AST and ALT in the control, T2DM, and obese + T2DM patient groups are shown in Figure 5. It was observed that the mean ± SD of AST and ALT levels in the T2DM and obese + T2DM groups were significantly increased (p < 0.0001) compared to the control group. The levels of AST and ALT were significantly higher (p = 0.0056) in the obese + T2DM patient group compared to the T2DM group.

**Figure 6:**
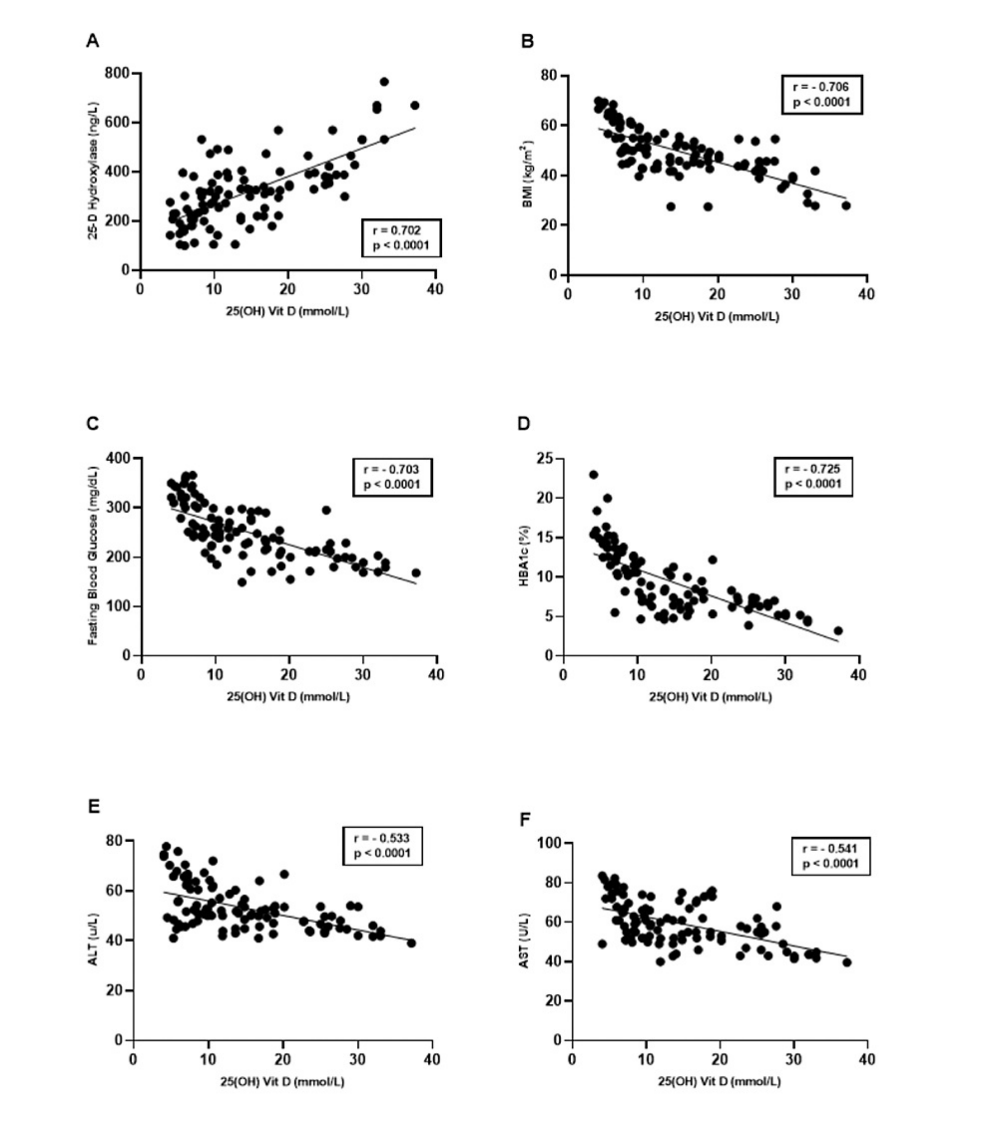
(A-F) Correlation coefficients between serum levels of 25 (OH) vitamin D and each of 25-D hydroxylase, BMI, fasting blood glucose, HbA1c%, ALT, and AST among patient groups. AST: aspartate transaminase, ALT: alanine transaminase.

In our study, we investigated the correlation coefficients between serum levels of 25(OH) vitamin D and each of 25-D hydroxylase, BMI, FBG, HbA1c%, ALT, AST, and lipid profile levels in both patient groups. A significant positive correlation was observed between the serum levels of 25(OH) vitamin D and 25-D hydroxylase (p < 0.0001, r = 0.702). Conversely, negative correlations were found between 25(OH) vitamin D and each of BMI (p < 0.0001, r = -0.706), FBG (p < 0.0001, r = -0.703), HbA1c% (p < 0.0001, r = -0.725), ALT (p < 0.0001, r = -0.533), and AST (p < 0.0001, r = -0.541), among all patient groups, as shown in Figure 6A-F.

**Figure 7:**
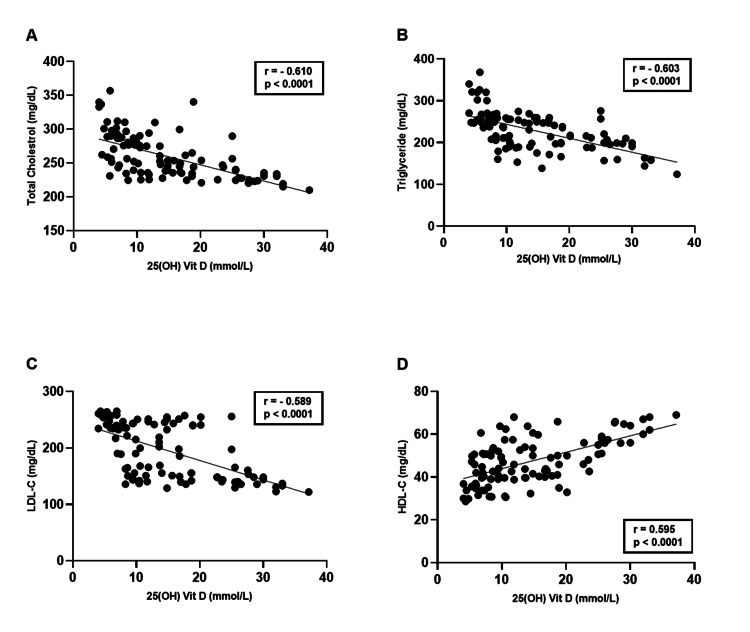
(A-D) Correlation coefficients between serum levels of 25(OH) vitamin D and lipid profile among patient groups. LDL: low-density lipoprotein, HDL: high-density lipoprotein.

Regarding serum levels of the lipid profile, negative correlations were found between the serum levels of 25(OH) vitamin D and TC (p < 0.0001, r = -0.610), TG (p < 0.0001, r = -0.603), and LDL-C (p < 0.0001, r = -0.589). A positive correlation was observed between the serum levels of 25(OH) vitamin D and HDL-C (p < 0.0001, r = 0.595) among patient groups (Figure 7A-D).

## Discussion

T2DM is a metabolic disorder characterized by high blood glucose levels due to insufficient insulin secretion or improper insulin use. Obesity is a major complication of T2DM, and vitamin D deficiency has been associated with an increased risk of developing T2DM [1]. In our present study, the results showed that the serum levels of 25(OH)D in the T2DM and obese + T2DM patient groups were significantly decreased (p < 0.0001) compared to the control group. The serum level of 25(OH)D in the obese + T2DM group showed a significant decrease when compared to the T2DM group.

A study conducted by Hussain et al. [13] revealed a significant statistical difference in 25(OH)D levels between patients with diabetes and those without. Bajaj et al. [14] reported that vitamin D deficiency and insufficiency were substantially more prevalent in individuals with T2DM than in healthy participants. These findings are consistent with those reported by other researchers, such as Payne et al. [15], who found that individuals with diabetes had lower levels of 25(OH)D than those without diabetes.

Additionally, we observed that the serum levels of 25 hydroxylase D were significantly decreased in the obese + T2DM and T2DM patient groups compared to the control group. The obese + T2DM group exhibited the greatest decrease. The main enzyme responsible for converting vitamin D to its active form, CYP2R1, is suppressed by obesity. However, a study has shown that gastric bypass surgery, which causes weight loss, can increase CYP2R1 expression in subcutaneous adipose tissue, indicating a potential recovery from suppression caused by obesity [16].

This study showed that the FBG levels in the diabetic and obese + T2DM groups were significantly increased compared to the control group. In the obese + T2DM group, the FBG levels were significantly higher compared to the T2DM patients' group. A cross-sectional study by Deng et al. [17] showed a significant increase in FBG levels among male and female obese individuals.

The present study showed that HbA1c% in the obese + T2DM and T2DM patient groups were significantly increased (p < 0.0001) compared to the control group. The obesity group was significantly higher compared to the diabetes group. In line with our findings, a retrospective study by Boye et al. [18] revealed that individuals with higher BMI levels had higher HbA1c% readings consistently across all years; their results were similar when analyzing different age groups. Zhao et al. [19] confirmed that HbA1c% is increased in patients with T2DM with 25-OH-D deficiency, and this deficiency may be a risk factor for high HbA1c% in patients with T2DM.

Our study investigated the lipid profile and showed a significant increase in the serum levels of TC, TG, and LDL-C with a significant decrease in serum HDL-C in both diabetic and obese +T2DM patient groups relative to the control group. In consistent with our study, Gharib et al. [20,21] studies found that the serum levels of TC, TG, and LDL-C in all patient groups were significantly higher than in the control group. The levels in diabetic obese +T2DM patients were significantly higher compared to diabetic patients. On the other hand, the levels of HDL-C in the diabetic and diabetic obese +T2DM groups were significantly decreased compared to the control group. Moreover, Shahwan et al. [22] found that patients with T2DM had hypercholesterolemia (>200 mg/dL) and hypertriglyceridemia (>150 mg/dL). Additionally, abnormal LDL-C levels (>130 mg/dL) were observed, and HDL-C levels were less than 40 mg/dL.

The results of our study in the obese +T2DM and T2DM patient groups showed that AST and ALT were significantly increased compared to the control group. In addition, the levels of AST were significantly higher in the obese +T2DM patient group than in the T2DM group. Similarly, the levels of ALT were significantly higher in the obese +T2DM patient group than in the T2DM group. In line with our study, Ali et al. [23] conducted a study to assess the relationship between serum liver enzyme activity and general and abdominal obesity in an urban Bangladeshi population; their results show that the mean level of serum ALT and AST was significantly higher in the obesity group than the normal BMI group. Horvath et al. [24] discovered that obesity can cause an increase in DNA methylation in liver tissue due to heightened oxidative stress, which can ultimately damage liver tissue. Moreover, It has been mentioned that the release of various proteins from visceral adipose tissues, including adipokines, resistin, leptin, visfatin, and tumor necrosis factor α, can impact liver function, potentially causing inflammation, cirrhosis, and hepatocellular cancer [25,26].

Consistent with our results, Alzahrani et al. [27] conducted a retrospective study that showed that serum AST and ALT levels were elevated in T2DM patients. Additionally, Islam et al. [28] mentioned that although the exact biological mechanism that explains the relationship between hepatic enzymes and the incidence of T2D is unclear, there are some possible explanations. One possibility is that higher levels of ALT and AST may indicate excess fat accumulation in the liver, a condition known as NAFLD. This condition is associated with metabolic syndrome, a cluster of cardiovascular risk factors that include insulin resistance, obesity, dyslipidemia, and T2D. NAFLD is also closely linked to obesity and visceral fat accumulation, a hallmark of insulin resistance syndrome. In fact, visceral adiposity is considered a major contributor to the development of T2D.

The present study indicates a positive correlation between 25(OH)D and 25-D hydroxylase. This finding is biologically plausible, as 25-D hydroxylase converts vitamin D into its active form. Any defects in this enzyme may result in abnormal vitamin D deficiency. Bakhamis et al. [29] studied and analyzed 27 patients from nine families who presented with low vitamin D and were not responding to usual treatment. A prospective cohort study was performed at King Faisal Specialist Hospital and Research Centre, Riyadh, Saudi Arabia; two mutations were identified through genetic testing: c.367+1G>A (in 12 of 27 patients) and c.768dupT (in 15 of 27 patients), which showed that these mutations were the cause of vitamin D deficiency and lack of response to treatment. Wamberg et al. [30] investigated whether the low levels of 25(OH)D in the serum of obese individuals could be due to altered vitamin D metabolism in adipose tissue. The researchers found that there were differences in the gene expression of vitamin D metabolizing enzymes between normal-weight and obese individuals; they specifically observed a 71 % decrease in the expression of the cytochrome P450 2J2 gene, which codes for the enzyme 25-hydroxylase.

Our study showed a negative correlation between serum levels of 25(OH)D and BMI. Several researchers have studied 25(OH)D deficiency in obese +T2DM patients. Recently, it has been demonstrated that there is an inverse relationship and strong correlation between 25(OH)D serum levels and BMI, most likely due to the volumetric dilution of vitamin D. This phenomenon occurs when the concentration of vitamin D in the body is lower due to the expansion of various compartments, such as fat, muscle, liver, and serum, in individuals with obesity. As these compartments increase in volume, the total amount of vitamin D remains relatively constant, but it becomes more dispersed throughout the expanded compartments. This dispersion leads to a lower concentration of vitamin D per unit of volume in the body, giving the impression of lower vitamin D levels [31]. Furthermore, the connection between obesity and low levels of circulating 25(OH)D may be due to the sequestration of vitamin D in adipose tissue and/or increased breakdown of vitamin D caused by the local action of 24-hydroxylase, which has been found in human fat tissue [32].

Our study demonstrated a significant negative correlation between 25(OH)D and FBG levels. In conformity with our results, Alrefai et al. [33] revealed that 25(OH)D levels were significantly lower in individuals with T2DM compared to the control group. Furthermore, individuals with T2DM and hypovitaminosis D had significantly higher FBG levels. The results also indicated a negative correlation between these patients’ FBG levels and 25(OH)D levels, as vitamin D appears to play a crucial role in enhancing insulin resistance in patients with T2DM and improving the sensitivity of peripheral insulin in individuals with impaired glucose tolerance. Our study also revealed a significant negative correlation between 25(OH)D and HbA1c%. In agreement with our results, Kostoglou et al. [34] showed a negative correlation between 25(OH)D levels and HbA1c% in patients with T2DM. This suggests that as 25(OH)D levels decrease, HbA1c% tends to increase in individuals with T2DM. It has studied and analyzed 28 patients with T2DM who received 100 micrograms (4000 IU) of vitamin D and 30 diabetic patients who received a placebo for two months and found a significant decrease in HbA1c% and increased insulin concentration. The action of vitamin D on the insulin receptor in beta cells is proposed as a potential mechanism linking vitamin D to diabetes. Vitamin D has the ability to increase the expression of the insulin receptor gene and facilitate glucose transport from the intestine [35].

Our study revealed a significant negative correlation between 25(OH)D and TC, TG, and LDL-C, and a significant positive correlation with HDL-C. Our results were consistent with Lupton et al. [36], whose study used a very large database of lipids, including 20,360 subjects. In multivariable-adjusted linear regression, individuals with deficient serum 25(OH)D had significantly lower levels of HDL-C and higher TC, TG, and LDL-C when compared with the optimal group. The phenomenon described in the statement indicates a negative relationship between BMI and 25(OH)D. As BMI is also linked to dyslipidemia, obesity could explain the observed connection between 25(OH)D and dyslipidemia. Additionally, there seems to be a correlation between the use of lipid-lowering medications and higher levels of 25(OH)D, implying that the observed association between low 25(OH)D levels and dyslipidemia might be attributed to patients with lipid imbalances not taking statin therapy. Another study conducted by Upreti et al. [37] in a placebo-controlled randomized pilot study on a group of individuals found that oral vitamin D supplementation was associated with improvements in glycemic control and other metabolic parameters. The study also observed significant reductions in TC and LDL levels among patients with T2DM.

The liver is responsible for the first metabolic transformation of vitamin D, which involves hydroxylation in position 25 to form 25(OH)D. The second transformation, 1α-hydroxylation, occurs in the kidneys. Because the liver is responsible for the crucial first stage of this process, impaired liver function may interfere with the proper vitamin D metabolism [38]. The results of our study revealed a significant negative correlation between serum 25(OH)D levels and the liver enzymes ALT and AST. In line with our study, a Korean study conducted by Cho et al. [39] found that a large proportion of adolescents with suspected NAFLD, defined as an increased ALT concentration (>30 U/L), had insufficient levels 25(OH)D levels (<20 ng/mL); therefore, adolescents with vitamin D deficiency were considered at increased risk of NAFLD. Furthermore, Apart from insulin resistance, the development of fatty liver disease is also attributed to inflammation and oxidative stress. These mechanisms are believed to be influenced by vitamin D deficiency, as vitamin D has been suggested to possess anti-inflammatory effects, particularly in macrophages. Furthermore, studies have indicated that vitamin D deficiency can impair T-lymphocyte function, increase inflammation by releasing pro-inflammatory cytokines, and elevate oxidative stress levels [40].

Limitations

The study involved a relatively small sample size of Saudi participants from a single clinic, which may limit the generalizability of the findings to other populations. Future studies with larger sample sizes, more diverse populations, and a non-diabetic obese control group could help to explore further the separate effects of obesity and diabetes on vitamin D levels and its potential role in the development of diabetes and obesity-related complications.

## Conclusions

This study provides evidence of a robust association between vitamin D and 25-D hydroxylase. Our findings suggest that the decrease of the enzyme is associated with a reduction in vitamin D levels in individuals with T2DM and obese diabetic patients. We also observed a significant correlation between vitamin D levels and each BMI, lipid profile, HbA1c%, FBG, AST, and ALT levels among these patient groups.
